# National Kidney Month — March 2013

**Published:** 2013-03-01

March is designated National Kidney Month to raise awareness about the prevention and early detection of kidney disease. In 2011, kidney disease was the ninth leading cause of death in the United States (1). More than 10% (>20 million) of U.S. adults aged ≥20 years have chronic kidney disease (CKD), and most of them are unaware of their condition (2,3). If left untreated, CKD can lead to kidney failure, requiring dialysis or transplantation for survival (2,4).

CDC’s Chronic Kidney Disease Initiative, in collaboration with partner agencies and organizations, has developed the CKD Surveillance System website (http://www.cdc.gov/ckd/surveillance) to document and monitor the burden of CKD and its risk factors in the United States. The website also provides the means for tracking progress toward achieving *Healthy People 2020* objectives to prevent, detect, and manage CKD and for evaluating, monitoring, and implementing quality improvement efforts by federal and nonfederal agencies.

Diabetes and high blood pressure are major risk factors for CKD, but controlling diabetes and blood pressure can prevent or delay CKD and improve health outcomes (2). Information about preventing and controlling kidney disease is available at http://www.nkdep.nih.gov. Information about CDC’s Chronic Kidney Disease Initiative is available at http://www.cdc.gov/ckd.
